# Vascular Resection in Perihilar Cholangiocarcinoma

**DOI:** 10.3390/cancers13215278

**Published:** 2021-10-21

**Authors:** Alejandro Serrablo, Leyre Serrablo, Ruslan Alikhanov, Luis Tejedor

**Affiliations:** 1 Section of Surgery, European Union of Medical Specialists, 1040 Brussels, Belgium; 2 HPB Surgical Division, Miguel Servet University Hospital, Zaragoza University, 50009 Zaragoza, Spain; 3 Medicine School, Zaragoza University, 50009 Zaragoza, Spain; leyre.sc22@gmail.com; 4 Division of Liver and Pancreatic Surgery, Moscow Clinical Research Center, 111123 Moscow, Russia; r.alikhanov@mknc.ru; 5 Department of Surgery, Punta Europa Hospital, 11207 Algeciras, Spain; tejedor@concadiz.es

**Keywords:** perihilar cholangiocarcinoma, vascular invasion in perihilar cholangiocarcinoma, biliary carcinoma, surgery in vascular involvement

## Abstract

**Simple Summary:**

In perihilar cholangiocarcinoma with vascular involvement, vascular resection to achieve margin-free status is being performed with increasing frequency despite controversial results. Morbidity, mortality, and overall survival are widely variable throughout the world. Vascular resections can include the portal vein alone, the hepatic artery alone, or combined resections. In some cases of locally advance disease, extended resections, such as hepatopancreatoduodenectomy or liver transplant, may be performed to achieve R0 status or a change to cure. The neoadjuvant treatment could help to achieve it. This article reviews and updates all treatment options in this setting.

**Abstract:**

Among the cholangiocarcinomas, the most common type is perihilar (phCC), accounting for approximately 60% of cases, after which are the distal and then intrahepatic forms. There is no staging system that allows for a comparison of all series and extraction of conclusions that increase the long-term survival rate of this dismal disease. The extension of the resection, which theoretically depends on the type of phCC, is not a closed subject. As surgery is the only known way to achieve a cure, many aggressive approaches have been adopted. Despite extended liver resections and even vascular resections, margins are positive in around one third of patients. In the past two decades, with advances in diagnostic and surgical techniques, surgical outcomes and survival rates have gradually improved, although variability is the rule, with morbidity and mortality rates ranging from 14% to 76% and from 0% to 19%, respectively. Extended hepatectomies and portal vein resection, or even right hepatic artery reconstruction for the left side tumors are frequently needed. Salvage procedures when arterial reconstruction is not feasible, as well as hepatopancreatoduodenectomy, are still under evaluation too. In this article, we discuss the aggressive surgical approach to phCC focused on vascular resection. Disparate results on the surgical treatment of phCC made it impossible to reach clear-cut conclusions.

## 1. Introduction

Altemeier in 1957 and Gerald Klatskin in 1965 were the first surgeons who described cholangiocarcinoma [1,2]. Between 50% and 70% of all cholangiocarcinomas are perihilar (phCC) or Klatskin tumors [3,4,5,6]. phCC is a highly unresectable malignancy because, despite being a slow growing tumor, its proximity to hepatic hilar structures leads to early vascular involvement, complicating surgical resection. Thus, most patients are diagnosed in an advanced stage of the disease which includes major vascular involvement. Surgical resection is the standard therapy for phCC and provides the only chance for cure in this disease. An aggressive surgical approach increases the number of resectable tumors that are initially regarded as unresectable [7], with 5-year survival rates (5-y SR) of 25–45% in R0 resections and of 0–23% in R1 resections [3,4,8,9]. Vascular resections (VRs) of the portal vein (PV), the hepatic artery (HA), or both add postoperative morbidity and mortality, although they achieve a higher R0 resection rate (i.e., microscopically negative margin), which is the most important factor to get increasing overall survival [3,9] (Table 1).

The aims of surgery in phCC are (1) to achieve the macroscopic removal of the tumor (VR increases the number of resected patients); (2) to satisfactorily restore bile flow to the gut; and (3) minimize postoperative liver failure or death. There are several surgical techniques to perform in these cases, since the extension of the resection depends on the radial extension of the tumor (leading to VR of the PV and/or HA), the longitudinal extension (if requiring a hepatopancreatoduodenectomy), or both (VR and hepatopancreatoduodenectomy) [3].

Advanced phCC requires extended liver resection and often VR, although margins may be affected in about one third of the patients [6]. Right-sided tumors, depending on their extension, often require extended right liver resections together with the PV, which is most optimally achieved with an en bloc resection or the Rex recess approach. Left-sided tumors frequently require extended left hepatectomy and often involve the contralateral PV or right HA, due to their proximity to the biliary bifurcation, therefore making their reconstruction necessary. Right HA involvement is more frequent. Arterial infiltration of the contralateral side of the planned hepatic resection is a contraindication to surgical treatment, though not in all centers. In patients with R0 resections, in histological analysis, the portal involvement is present in 20–30%, and its preoperative identification is achieved with an accuracy of 85% [14,18,20,27,31,33,34].

**Figure 1:**
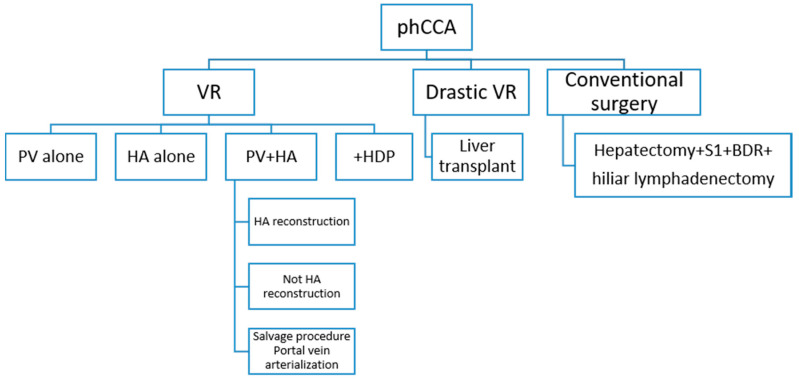
Vascular resections options. phCCA: perihilar cholangiocarcinoma; VR: vascular resection; PV: portal vein; HA: hepatic artery; HPD: hepatopancreatectoduodenectomy; S1: segment 1; BDR: biliary duct resection.

The conventional surgical technique for the treatment of phCC is right or left hepatectomy, plus segment 1 resection, plus biliary duct resection, plus hilar lymphadenectomy. To this technique, a PV resection alone, a HA resection alone, both (HA resection may be followed or not by a HA reconstruction or a PV arterialization), or a pancreatoduodenectomy can be added. Liver transplantation is also a possible treatment considered as a drastic vascular resection (Figure 1) [3,35,36,37].

If a consensus is not achieved on the surgical treatment of colorectal liver metastases, in the case of the treatment of pHCC, the final picture is even more complex [38]. Controversies arise regarding “on demand” or “elective” PV resection, HA resection in the remnant liver, left or right extended hepatectomy in Bismuth type IV, and liver transplant. Difficulties in analyzing the available data and the ability to draw clear conclusions on the efficacy of these treatments are due to the use of different classifications, both surgical (Bismuth and Corlette, 1975) and oncological (extension of tumor within the biliary tree, vascular invasion, lobar atrophy, and metastatic disease); heterogeneity of data and series, since many large series are limited to very specific areas; the number of different preoperative, postoperative, and histological staging classifications; large differences in the range of results; differences in neoadjuvant chemotherapy and radiotherapy protocols used in the last decades, and significant differences between Western and Eastern countries (even within the same country) in the management of vascular involvement.

Several limitations should be considered when interpreting data, according to Liang’s study. Although we only selected high-quality studies, all of them were predominantly retrospective in nature and, as such, there may be inherent selection bias. Additionally, heterogeneity in the selection of patients may have led to selection bias. Finally, some prognostic factors displayed significant heterogeneity [39].

**Figure 2:**
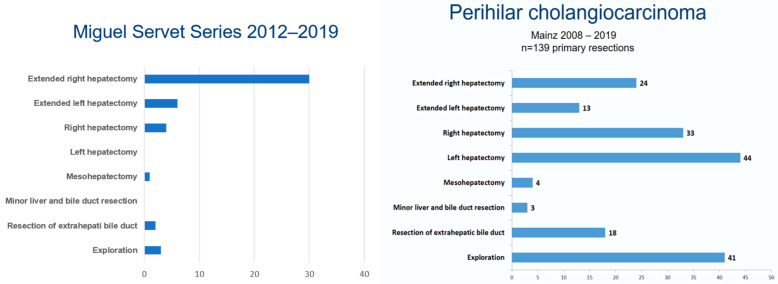
Miguel Servet/Mainz Series.

There are notable differences between Western and Eastern countries in the use of PV embolization, PV resection, HA resection and even in the future remnant liver volume (FRL). All of them, except FRL, are more frequently performed in Eastern countries, with reported morbidities and mortalities lower than in Western ones [40]. Even in the same zone there are several differences too. Figure 2 shows great differences between two European hospitals.

In Europe, in experienced centers the 90-day postoperative mortality is more than 10%. Around 48% of the patients die from post-hepatectomy liver failure [41,42]. In the largest center in Asia, the overall mortality was 4.7% for the period 1977–2010, decreasing sharply from 11.1% to 1.4% for the periods 1977–1990 and 2006–2010, respectively, even after including patients with more locally advanced disease during the latter period [6]. The presence of Bismuth type IV phCC (involving both the right and left intrahepatic ducts) is no longer an absolute contraindication to complete resection, since it is associated with an overall survival (OS) similar to that of patients with less extensive biliary extension [43]. Moreover, resection and reconstruction of the PV and HA are increasingly performed [9,33].

In an Australian study, there were a higher mortality and morbidity in the VR patient group, and these two rates increased when HA resection was performed [33]. The overall series had 50.8% morbidity and 7.2% mortality, but VR was only done in 29.6% of the cases. The authors concluded that PV invasion did not preclude the curative resection and that it should be performed in case of PV involvement.

## 2. Portal Vein Resection

True PV invasion in phCC is difficult to determine preoperatively. When computed tomography is analyzed, vessel constriction, loss of a clear plane, and occlusion are considered evidence of venous invasion.

Left PV resection is not a technically complex procedure. Usually, end-to-end anastomosis is possible with or without graft interposition, autologous or not. Grafting is necessary when the length of resection is more than 5 cm however, since the left PV has a long extrahepatic path and there is an easy access to the vein into the umbilical fissure, it is almost always possible to avoid grafting [43,44,45]. Generally, there is not much difference in diameter between both ends, and it is possible to perform a standard anastomosis.

The right PV is short and bifurcates early in its course. The limits of right PV resection depend on whether the first branches can be controlled with clamps. A Y graft may be necessary. There are discrepancies in the diameters of the main PV and the right branches (especially between the right posterior sector branch and the main PV) [43,46]. The Rex recess approach includes a right hepatectomy with en bloc resection of the hepatoduodenal ligament and PV reconstruction to the left portal vein at the Rex recess [43,45].

In general, PV bifurcation should be resected only when tumor adherence or infiltration has been detected or when it seems to be invaded. The Nehaus team’s practice of PV resection a priori has not yet been validated. In 2019, Higuchi et al. found that the absence of neoplastic invasion of the vein in histological analysis was a good prognostic factor compared with the presence of high dysplasia or in situ tumor [47]. In 2018, van Vugt et al. concluded that both unilateral and main HA involvement are independent poor prognostic factors for overall survival, whereas PV involvement is not [48]. Actual rates of venous invasion on histopathological examination after resection vary from 21% to 80% according to Abbas’s meta-analysis [33].

The point of controversy regarding portal vein resection is whether it is performed systematically or on demand, based on the radiological and intraoperative findings [49]. In 2012, Nehaus compared two groups of 50 patients each, one with en bloc resection of the PV and the other with only major hepatectomy. The first group had a better survival rate than the second group. Although evaluation of short-term results failed to reveal any association between combined PV resection and a high postoperative complication rate, a correlation between PV resection and a higher mortality was identified [49]. The mean mortality rate of combined PV resection is lower in studies with larger sample sizes, and was also lower in studies published after 2008. Liver failure was and is the main cause of postoperative mortality, although the management of jaundice with percutaneous drainage and improvements in anesthesia management have decreased mortality in the last decade.

Ebata et al. found that macroscopic portal vein invasion was a poor prognostic factor [20]. In the Netherland group, Rassam et al. in 2018 showed that 20% of their cases had PV resection, with a 44.3% 5-y SR, similar to 43% in the Berlin group of R0 resections using unconditional en bloc resection [50].

We can conclude that, currently, on demand PV resection has the same survival rate as resection en bloc, so the decision on whether to resect the PV or not should be made in the operating theatre. PV resection increases the survival rate, but has significantly high rates of morbidity and mortality depending on centers. De Jong et al. analyzed 305 patients, with PV resection performed in 16.7% of them. Thirty- and ninety-day mortality was more that four-fold higher in this group, compared with the non-vascular resected group [9].

## 3. Hepatic Artery Resection

The right hepatic artery is closely associated with posterior or anterior surface of the biliary confluence and is often involved by tumors, but its preservation is utterly important for the remnant liver after left or extended left hepatectomies. R0 resections requiring resection and reconstruction of an involved artery are associated with high morbidity and mortality [33]. The left hepatic artery is infrequently involved by tumors because it runs well away from the biliary confluence, therefore in extended right hepatectomy it is rare to need hepatic artery reconstruction. The rate of positive involvement in the resected hepatic artery is lower than in the resected portal vein (PV 47.1% and HA 40%) [46].

Arterial resection and reconstruction are usually performed in a left-sided resection (IIIb) for anatomical reasons. Most reports show dismal results: Gerhads et al. reported a 55.6% mortality; Ota et al. stated a 46.9% mortality, but their series was about HPD; Yamanaka et al. reported a 10% mortality but 90% morbidity; Shimada et al. reported a mortality with HA resection and reconstruction of 13.3% vs. 8.3% without it; Sakamoto et al. reported 0% mortality, and Miyazaki et al. stated a 33% mortality and 0% 3-y SR in patients with HA resection [21,25,51,52,53,54].

The inflection point of the current improvement in surgical techniques lies in the knowledge derived from living donor liver transplant techniques, since they have been key to improving these data. After 2010, outcomes began to be better than before (Table 2).

In a series from 2010 to 2020 with 425 patients, 37.4% had an extended left hepatectomy, 1.2% an extended right hepatectomy, and 65.1% a combined PV resection. Morbidity was 47%, mortality was 7.5% and the 5-y SR was 27.2%. All these data come from the higher quality Asian groups.

Nagino et al. did not find statistically significant differences associating VR or pancreatoduodenectomy. However, despite these aggressive procedures, the circumferential margin was positive in 34% of the patients and 50% of them had nodal involvement, although they reported a 1, 3, and 5-y SR of 78.9%, 36.3%, and 30.3%, respectively [62,63]. They defended HA resections when they analyzed unresected patients in comparation with arterial and/or portal vein resections.

Some surgical refinements have been proposed. The De Santibañes et al. and Iida et al. groups gave the surgeon the possibility of creating a satisfactory anastomosis before the resection began and the possibility of abandoning the procedure if it was not feasible [64,65]. As Bismuth type IIIB often requires a major left hepatic resection and invasion of the right HA usually contraindicates the procedure, they proposed performing a HA reconstruction between the posterior branch of the right HA and the left HA as the first surgical step, before transection of the parenchyma and the hilar resection. Uchiyama et al., in an excellent technical article, tried to standardize PV resection and HA resection in extended left hepatectomy, although perhaps this complex surgery should be centralized in specific groups [66].

We can decide not to perform a right HA reconstruction. This is possible when the right HA or one of the right hepatic arteries come from the superior mesentery artery, or when, in patients undergoing left-sided resections involving RHA, the liver is minimally mobilized to preserve the collaterals. Some surgeons prefer to embolize the proper HA, or the left or right HA, to stimulate growth of collateral arteries, but this procedure carries a great risk of ischemia [67,68,69].

van Vugt et al. concluded that HA involvement (one or both) was a poor prognostic factor but that the PV involvement was not [48]. Govil et al. concluded that a way to perform a safer and potentially curative surgery in extended left liver resections by phCC is to have experience in performing safe arterial resection and reconstruction [70]. This, in turn, increases the resectability rate for pHCC, particularly for Bismuth–Corlette type IV tumors.

If both the PV and HA are involved, resection and reconstruction of both can be performed. PV anastomosis should be performed first if both are resected [62].

The complications of arterial resections and reconstructions are bleeding and thrombosis with deleterious effects to liver parenchyma, pseudoaneurysms, or aneurysms.

## 4. Vascular Resection and Hepatopancreatoduodenectomy

We can find deeply differing results between Eastern and Western countries regarding survival rate. Nagino et al. in 2021 showed a 5-y SR of 37% but Souza et al. in 2021 reported a lower SR and a 17% 90-day mortality [63,70,71].

In Nagino’s series, patients were a mean of 60 years old. Most of the combined VRs and HPDs were performed with extended left hepatectomy [63]. PV reconstruction was performed with external iliac venous graft and HA reconstruction with end-to-end anastomosis except in two cases (one with portal vein arterialization and one using the radial artery). Despite this huge surgical tour de force, R1 resection was present in 45% of the cases, although the 5-y SR was 37%. In the study by Ebata et al. in 2014, hepaticopancreatoduodenectomy combined with VR was a poor prognostic factor, together with histological status [36].

Due to the complexity of the surgery and its high morbidity and mortality, in addition to the dispersion of the data and the concentration of the series in a few centers, no conclusion can be drawn.

## 5. Portal Vein Arterialization (PVA)

PV arterialization has been used as a salvage procedure when arterial reconstruction fails during surgery, with a success rate of around 60%. In the series from the Paul Brousse Hospital, there were 4 intrahospital deaths and 10 deaths between 2 and 30 months of follow-up out of 16 patients. Complications related with this procedure are hyperbilirubinemia and hemorrhage due to portal hypertension. At times we have needed to urgently embolize this shunt due to uncontrolled hemorrhage [72]. We performed this procedure during extended left hepatectomy with curative intention except in one patient (25%), in whom the shunt was performed during the postoperative course as an emergency surgery, with a mortality of 50%. If the liver does not totally mobilize, the hepatic artery could not be reconstructed with uncertain results. Furthermore, portal vein arterialization should be closed by interventional radiologists to avoid right heart failure (Table 3).

Making conclusions in this setting is very difficult, because PV arterialization is usually an unplanned approach, even a rescue procedure and, although it is a technique that hepato-pancreato-biliary surgeons must know, its results are difficult to predict.

## 6. Liver Transplantation (LT)

Liver transplantation is the most radical procedure in terms of vascular resections. Under strict conditions, LT may be offered in unresectable phCCs in patients with (1) a malignant appearing stricture and at least one of the following: malignant cytology or histology; CA-19.9 >130 U/mL without cholangitis; polysomy on fluorescence in situ hybridization; a mass on cross-sectional imaging ≤3 cm and no extrahepatic disease; (2) a cancer located primarily above the cystic duct; and (3) an unresectable cancer de novo phCCA or cancer arising in the setting of primary sclerosing cholangitis [73].

Loveday et al. reported one- and two-year post-transplant overall survival of 83.3% and 55.6%, respectively, in intention-to-treat patients [74]. In the European Liver Transplant Register’s experience, Mantel et al. reported a 59% 5 y-SR in patients within the Mayo Clinic criteria and only 21% in those beyond it. Therefore, the authors advocated that the selection criteria should be within the Milan protocol [75].

In 2018, Ethun et al. defended the premise that patients with resectable phCC would obtain superior survival with liver resection compared to liver transplantation had changed. The authors, in their prospective observational study, sought to validate the results after following the Mayo Clinic protocol for orthotopic LT for “unresectable disease”, and to compare them with the results after hepatectomy for “resectable” phCCA [76]. The transplant group achieved a much better 5-y SR with 64% compared to 18% in the group that was resected (*p* < 0.001), despite having inoperable or more advanced disease [76].

In the Transplant Oncology Consensus Conference 2020, the agreed conclusions were that LT for phCC is an acceptable indication, that patients should undergo neoadjuvant chemoradiation prior to LT, that the inclusion criteria for LT should be based on the Mayo Clinic criteria, and that, due to organ allocation issues, living donor liver transplant, if possible, is the preferred option.

## 7. Conclusions

We can conclude (1) that advanced pHCC requires an extended hepatic resection and frequently a vascular resection too; (2) extended right hepatectomy in right-sided tumors is likely to need PV resection with end-to-end anastomosis or a Rex recess approach; (3) PV resection increases morbidity and mortality but achieves R0 resection more frequently and should be performed “on demand”; (4) extended left hepatectomy in left-sided tumors is likely to need PV resection as well as HA resection to achieve an R0 resection; (5) arterial reconstruction causes more morbidity and mortality and its oncological benefits are unclear; (6) HPD with VR should be performed in high level centers and in very select patients; (7) PV arterialization is a salvage procedure with uncertain outcomes; and (8) liver transplant could be key to rescuing more patients with vascular involvement within the Mayo Clinic/Toronto Protocol.

## Figures and Tables

**Table 1:** Vascular resection in phCC.

Study	Year	Patients, *n*	Vascular Resection, *n*	Morbidity %	Mortality %	5-y SV	R0 %	R1 %	R0 with VR%
Lygidakis et al. [10]	1988	13	7	NA	15	NA	NA	NA	46
Edmond et al. [11]	1989	13	5	69	15	NA	NA	NA	46
Klempnauer et al. [12]	1997	125	41	29.8	9.9	28	26	6.8	73
Magriaga et al. [13]	1998	28	9	32	14	8	11	0	50
Neuhaus et al. [14]	1999	66	23	56	3	22	42	9	61
Lee et al. [15]	2000	111	29	22	6.3	24	NA	NA	77
Nimura et al. [16]	2000	142	43	48.6	9	25	26	16	61
Nagino et al. [17]	2001	105	33	81	9.5	NA	NA	NA	NA
Munñoz et al. [18]	2002	28	10	25	3	23	NA	NA	NA
Neuhaus et al. [19]	2003	133	NA	NA	NA	NA	38	18	NA
Ebata et al. [20]	2003	160	52	84	9.6	37	NA	NA	NA
Shimada et al. [21]	2003	39	15	71	6.7	56	50	10	50
Kondo et al. [22]	2004	42	14	48	0	NA	NA	NA	95
Hemming et al. [23]	2005	53	23	40	9	35	45	0	80
Baton et al. [24]	2007	59	5	42	5	20	28	6	67
Miyazaki et al. [25]	2007	161	43	39	7	NA	36	0	36
Hidalgo et al. [26]	2008	44	17	66	6.8	41	45	26	45
Song et al. [27]	2009	259	51	54	9.6	29.3	29.3	17	71.8
Igami et al. [28]	2010	298	111	43	2	42	52	32	66
Young et al. [29]	2010	51	21	75	8	20	40	2	57
Miyazaki et al. [30]	2010	107	25	NA	2	NA	33	21	59
Nagino et al. [31]	2010	261	50	54	2	30	40.7	0	54
Hemming et al. [32]	2011	95	42	36	5	43	50	0	84
All	24 years	2393	30.4	50.8	7.2	30.2	37	10.2	62

phCC: perihilar cholangiocarcinoma; R0: R0 resection; VR: vascular resection; R1: R1 resection; NA: not applicable.

**Table 2:** Arterial resection in phCC.

Author	Year	Cases	Hepatic Left Trisectionectomy	Hepatic Right Trisectionectomy	Simultaneous PVR	Morbidity (%)	Mortality (%)	5-y SV
Nagino et al. [31]	2010	50	26 (52%)	0	50 (100%)	54	2	30
Wang et al. [55]	2015	24	0	0	18 (75%)	42	4	25
Matsuyama et al. [56]	2016	44	22 (50%)	0	24 (55%)	66	9	22
Noji et al. [57]	2016	28	7 (25%)	0	23 (82%)	57	4	26
Peng et al. [58]	2016	26	0	0	2 (8%)	19	8	31
Hu et al. [59]	2018	63	12 (19%)	3 (1%)	35 (56%)	19	3	22
Schimizzi et al. [60]	2018	12	0	1 (8%)	2 (17%)	67	8	-
Higuchi et al. [47]	2018	19	1 (5%)	0	12 (63%)	47	16	16
Kotenko et al. [61]	2019	13	NA	NA	13 (100%)	NA	9.3	18.8
Mizuno et al. [62]	2020	146	86 (59%)	1 (1%)	100 (68%)	51	4	27
All		425	154 (37.4%)	5 (1.2%)	277 (65.1%)	0.47	7.5	27.2

PVR: portal vein resection; phCC: perihilar cholangiocarcinoma; NA: not applicable.

**Table 3:** Portal vein arterialization.

Hospital	Age/Sex	Hepatobiliary Surgery	Primary Procedure	Portal Vein Arterialization	Timing	Type
		Indication for Surgery		Indication for PVA and Timing		
Paul Brousse	61/M	Hilar cholangiocarcinoma (Klatskin type IIIB)	Left extended hepatectomy (including segments I, V, VIII)	For curative resection (RHA involvement)	During LR	CHA to PV
Miguel Servet	56/M	Hilar cholangiocarcinoma (Klatskin type IIIB)	Left extended hepatectomy (including segments I, V, VIII)	For HA thrombosis (LHA to RPHA). Salvage procedure	20th POD	PHA to PV
Miguel Servet	71/F	Hilar cholangiocarcinoma (Klatskin type IIIB)	Left extended hepatectomy (including segments I, V)	For curative resection (RHA involvement)	During LR	PHA to PV
Miguel Servet	68/F	Hilar cholangiocarcinoma (Klatskin type IIIB)	Left extended hepatectomy (including segment I)	For curative resection (RHA involvement)	During LR	PHA to PV
Miguel Servet	74/F	Hilar cholangiocarcinoma (Klatskin type IIIB)	Left extended hepatectomy (including segments I, V, VIII)	For postoperative complication with pseudoaneurysm 21 POD. Rescue after hepatic artery reconstruction	21st POD	CHA to PV
Shizuoka General	64/M	Hilar cholangiocarcinoma (Klatskin type IIIB)	Extended left LR + PD with RHA excision/ reconstruction	Post op HAT in reconstructed artery	1 POD	Mesenteric vascular branches (ileal)
Shizuoka General	72/F	Hilar cholangiocarcinoma (Klatskin type IIIa)	Extended right LR	Postoperative ligation of CHA following HAP rupture (day 6) causing massive liver necrosis	7th POD	First PVA—mesenteric vascular branches (ileocecal)
Shizuoka General	65/M	Hilar cholangiocarcinoma (Klatskin type IIIB)	PD, extended left LR, excision of anterior branch RHA	For curative surgery (pre-emptive shunt)	5 days before major resection	Mesenteric vascular branches (ileal)
Tsuruga National Hospital	NA	Hilar cholangiocarcinoma	Left extended LR with HAP excision	For curative surgery	During LR	GDA to PV
Hokkaido University	56–81	Hilar cholangiocarcinoma (6)	Major liver resection with en bloc HA resection	For curative surgery	During LR	GDA or CHA to PV
St James’s University	54/M	Hilar cholangiocarcinoma	LR	For curative surgery	During LR	GDA to PV
St James’s University	51/F	Hilar cholangiocarcinoma	LR	For curative surgery	During LR	RHA to PV
General Hospital of Chinese People’s Liberation Army	50–54	Hilar cholangiocarcinoma (3)	LR	For curative surgery	During LR	HA to PV (with calibration)
West China Hospital	55/M	Hilar cholangiocarcinoma	LR	For curative surgery	During LR	GDA to PV

M: male; F: female; PVA: portal vein arterialization; RHA: right hepatic artery; LHA: left hepatic artery; RPHA: right posterior hepatic artery; POD: postoperative days: HAT: hepatic artery thrombosis; CHA: common hepatic artery; PHA: proper hepatic artery; LR: liver resection; PD: pancreatoduodenectomy; GDA: gastroduodenal artery; PV: portal vein; HA: hepatic artery.
